# Lignin Hydrolysis and Phosphorylation Mechanism during Phosphoric Acid–Acetone Pretreatment: A DFT Study

**DOI:** 10.3390/molecules191221335

**Published:** 2014-12-18

**Authors:** Wu Qin, Lingnan Wu, Zongming Zheng, Changqing Dong, Yongping Yang

**Affiliations:** National Engineering Laboratory for Biomass Power Generation Equipment, School of Renewable Energy Engineering, North China Electric Power University, Beijing 102206, China

**Keywords:** lignin, lignocellulose, biomass, pretreatment, DFT

## Abstract

The study focused on the structural sensitivity of lignin during the phosphoric acid–acetone pretreatment process and the resulting hydrolysis and phosphorylation reaction mechanisms using density functional theory calculations. The chemical stabilities of the seven most common linkages (β*-O-*4, β-β, 4*-O-*5, β-1, 5-5, α*-O-*4, and β-5) of lignin in H_3_PO_4_, CH_3_COCH_3_, and H_2_O solutions were detected, which shows that α*-O-*4 linkage and β*-O-*4 linkage tend to break during the phosphoric acid–acetone pretreatment process. Then α*-O-*4 phosphorylation and β*-O-*4 phosphorylation follow a two-step reaction mechanism in the acid treatment step, respectively. However, since phosphorylation of α*-O-*4 is more energetically accessible than phosphorylation of β*-O-*4 in phosphoric acid, the phosphorylation of α*-O-*4 could be controllably realized under certain operational conditions, which could tune the electron and hole transfer on the right side of β*-O-*4 in the H_2_PO_4_^−^ functionalized lignin. The results provide a fundamental understanding for process-controlled modification of lignin and the potential novel applications in lignin-based imprinted polymers, sensors, and molecular devices.

## 1. Introduction

Lignin is a complex polymer of aromatic alcohols mostly derived from wood [1,2] and accounting for 15%–20% of lignocelluloses [3]. Lignin plays important roles in resistance to pests and diseases, and nutrient transport with sunlight and frost stress responses [4]. The advantages of using organosolv lignin in the production of phenolic resins include a reduction in the consumption of formaldehyde and enhancement of the resulting resins’ wear behavior [5]. Lignin derivatives have been used to produce epoxy resins, polyurethane and isocyanurate resins [6]. Lignin can be blended with natural polymers (e.g., starch), acts as an antioxidant or flame-retardant agent, and absorbs UV radiation [4]. The incorporation of epoxy-modified lignosulfonate into a polypropylene/polyethylene compatibilizing agent produced a polymer with good thermal, physico-mechanical, and surface properties [7]. Lignin could be used as a binder in mortar and construction systems and for metal sequestration in solutions. The sulfur-free lignin isolated from pulping has been tested as a mortar additive [8]. It was found to be comparable in performance to commercially available lignins, such as Organocell, Alcell, and Curan100. The industrial lignins obtained from different sources exhibit high antioxidant capacity over a range of concentrations, suggesting the potential use of lignins in cosmetic and topical medical formulations [9]. The PLA/APP/PER composite containing lignin shows lower limiting oxygen index values, but it is still commercially acceptable. The UL-94 ratings are superior to that containing pentaerythritol [10]. For these important applications of lignin, research turns to how obtain lignin from biomass.

**Figure 1:**
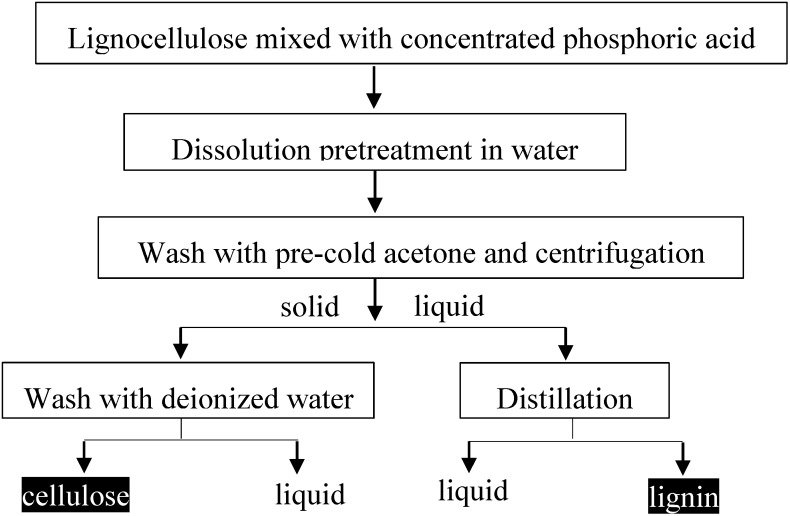
The phosphoric acid–acetone pretreatment process.

Recently, the phosphoric acid–acetone pretreatment process represents one of the most efficient methods to separate lignin and cellulose from lignocellulose, where bonds between lignin and cellulose break and cellulose dissolves into H_3_PO_4_, then the dissolved cellulose sediments in acetone while the lignin dissolves in the acetone [11,12,13,14]. Figure 1 shows the principle of the phosphoric acid–acetone pretreatment process.

In these processes, H_3_PO_4_ is a weak triprotic acid, non-toxic and inexpensive compared with other mineral acids. Cellulose can be dissolved in phosphoric acid more simply, faster and at lower temperatures. The hydrogen ions from H_3_PO_4_ could be easily diffused into cellulose [15]. Two phenomena occur after dissolution: the first one is that an esterification reaction that happens between the hydroxyl groups of cellulose and H_3_PO_4_ to form cellulose phosphate, and a competition of hydrogen-bond formation among the remaining hydroxyl groups on cellulose chains, a hydrogen ion and a water molecule; the second case is that the pretreated cellulose changes into amorphous cellulose after phosphoric acid pretreatment [11]. The amorphous regions bundle with the remaining crystalline structure of cellulose, resulting in the uneven and rough molecular surface. Moreover, the pretreated cellulose reaches its adsorption equilibrium in a relatively shorter time, suggesting that H_3_PO_4_ pretreatment can accelerate the enzyme adsorption rate and enhance the subsequent hydrolysis process. However, limited attention has been played to the behavior of lignin in the phosphoric acid-acetone pretreatment process. The detailed interaction between lignin and solution, the related reaction mechanism and the resulting modified properties of the lignin remain unknown.

To address these fundamental questions, we used models for the seven most common linkages in lignin (accounting for approximately 87% of linkages in softwood lignin [16]) to detect the chemical stability of these seven linkages to reveal in details the sensitivity of lignin, the hydrolysis and phosphorylation occurring to the vulnerable linkages, and the process-tuned properties of the modified lignin.

## 2. Results and Discussion

### 2.1. Decomposition of Lignin in Various Solutions

**Figure 2:**
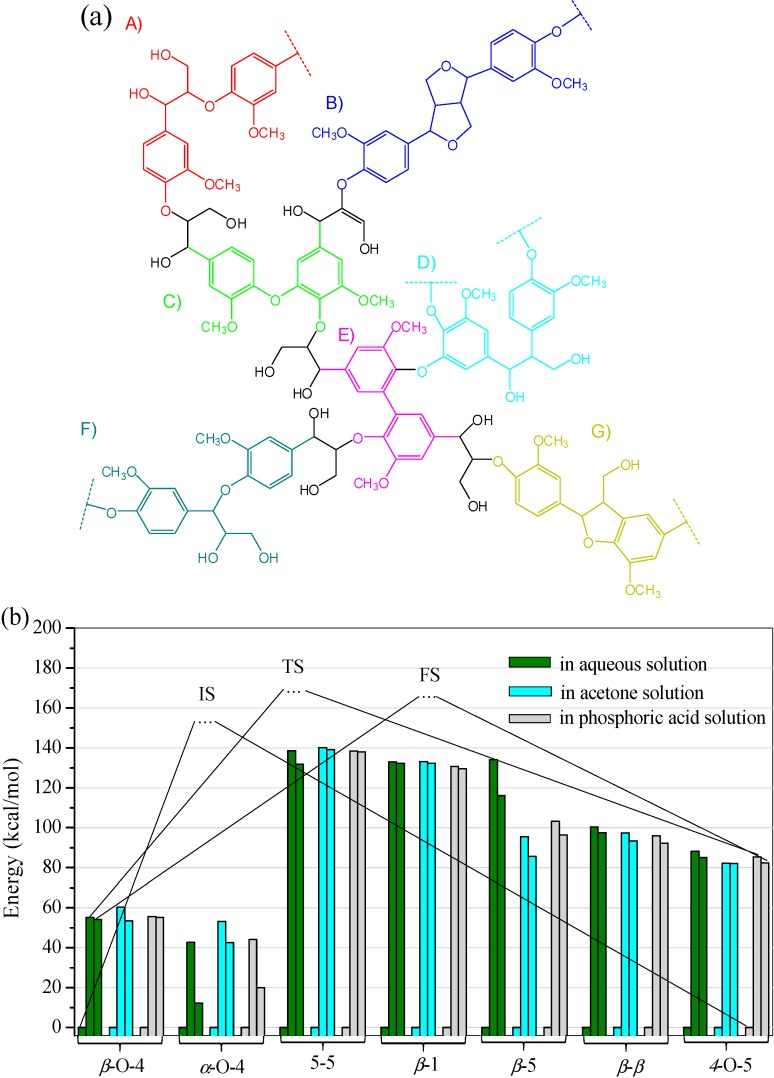
(**a**) Stable lignin fragment highlighting the linkages studied in this work: (A) β*-O-*4; (B) β-β; (C) 4*-O-*5; (D) β-1; (E) 5-5; (F) α*-O-*4; and (G) β-5; (**b**) Potential energy profiles for the decomposition reaction of different linkages in H_3_PO_4_, CH_3_COCH_3_, and H_2_O solutions.

Models of the model lignin with the most common linkages (β*-O-*4, α*-O-*4, 5-5, β-1, β-5, β-β, and 4*-O-*5) were built in reference to the work of Beste [17]. Figure 2 shows the stable configuration of the model lignin and the calculated potential energy profiles for the decomposition of the seven linkages in H_2_O, CH_3_COCH_3_, and H_3_PO_4_ solutions. The reactions start from the optimized geometries of β*-O-*4, α*-O-*4, 5-5, β-1, β-5, β-β, and 4*-O-*5 in these different solutions, respectively. The energy barrier (*E*_a_) for β*-O-*4 decomposition in H_2_O, CH_3_COCH_3_, and H_3_PO_4_ solutions is 55.2 kcal/mol, 60.4 kcal/mol, and 55.5 kcal/mol, respectively, while the reaction energy (*E*_r_) is 54.1 kcal/mol, 53.544 kcal/mol, and 55.2 kcal/mol. *E*_a_ and *E*_r_ for α-*O*-4 decomposition in H_2_O, CH_3_COCH_3_, and H_3_PO_4_ solutions is 42.8 kcal/mol and 12.2 kcal/mol, 53.2 kcal/mol and 42.5 kcal/mol, 44.1 kcal/mol and 19.8 kcal/mol, respectively. *E*_a_ and *E*_r_ for 5-5 decomposition in H_2_O, CH_3_COCH_3_, and H_3_PO_4_ solutions is 138.6 kcal/mol and 131.9 kcal/mol, 140.1 kcal/mol and 139.2 kcal/mol, 138.3 kcal/mol and 138.0 kcal/mol, respectively. *E*_a_ and *E*_r_ for β-1 decomposition in H_2_O, CH_3_COCH_3_, and H_3_PO_4_ solutions is 133.0 kcal/mol and 132.4 kcal/mol, 133.1 kcal/mol and 132.2 kcal/mol, 130.7 kcal/mol and 129.6 kcal/mol, respectively. *E*_a_ and *E*_r_ for β-5 decomposition in H_2_O, CH_3_COCH_3_, and H_3_PO_4_ solutions is 100.3 kcal/mol and 97.5 kcal/mol, 97.4 kcal/mol and 93.3 kcal/mol, 95.8 kcal/mol and 92.2 kcal/mol, respectively. *E*_a_ and *E*_r_ for β-β decomposition in H_2_O, CH_3_COCH_3_, and H_3_PO_4_ solutions is 134.1 kcal/mol and 116.1 kcal/mol, 95.6 kcal/mol and 85.6 kcal/mol, 103.2 kcal/mol and 96.4 kcal/mol, respectively. *E*_a_ and *E*_r_ for 4*-O-*5 decomposition in H_2_O, CH_3_COCH_3_, and H_3_PO_4_ solutions is 88.2 kcal/mol and 84.9 kcal/mol, 82.2 kcal/mol and 82.0 kcal/mol, 85.4 kcal/mol and 82.4 kcal/mol, respectively. The results imply that energetically 5-5, β-1, β-5, β-β, and 4*-O-*5 are rather chemically stable during the phosphoric acid–acetone pretreatment process, which can maintain the structural integrity and the yield of lignin during the pretreatment process [18]. However, partial structural damage happens to lignin due to the relative vulnerability of β*-O-*4 and α*-O-*4, and as mentioned before that β*-O-*4 is one of the main cleavage modes [19] and the dissociation enthalpy of α*-O-*4 is small (40–44 kcal/mol in gas phase) [20]. Interestingly, the breakage of α*-O-*4 linkages resulted in relatively permanent damage to lignin, since it is relatively difficult for the reverse process of the decomposition to happen due to the higher activation energy ($\overset{↼}{E_{a}}$) for the reverse reaction in H_2_O and H_3_PO_4_ solutions, compared to the decomposition process of β*-O-*4 linkage. These results are similar to the work reported by Kim *et al.* [21].

The charge density difference of the system at the critical points, *i.e.*, initial state (IS), transition state (TS), and final state (TS) of the decomposition reaction of β*-O-*4, α*-O-*4, 5-5, β-1, β-5, β-β, and 4*-O-*5, respectively, is further discussed. The different density isosurfaces for the β*-O-*4, α*-O-*4, 5-5, β-1, β-5, β-β and 4*-O-*5 linkage are symmetric at IS. However, at TS and FS, the electron density is enriched at the O atom of the β*-O-*4, α*-O-*4 and 4*-O-*5 linkage, respectively, while the electron density is depleted at the corresponding C_β_, C_α_, and C_4_ atom; for the β-1 and β-5 linkages, the electron density is enriched at one C_β_ atom and depleted at the C_1_ atom and C_5_ atom, respectively; also for the 5-5 and β-β linkages, the electron density is enriched at one C atom and depleted at the other C atom, respectively. The partial electron transfer through the reaction resulted in an unsymmetrical electron density population, which implies that the decomposition of β*-O-*4, α*-O-*4, 5-5, β-1, β-5, β-β, and 4*-O-*5 in these solutions will proceed via a heterolytic, unzipping mechanism, as described in the work of Sturgeon *et al.* [22].

### 2.2. Phosphorylation of Lignin

**Figure 3:**
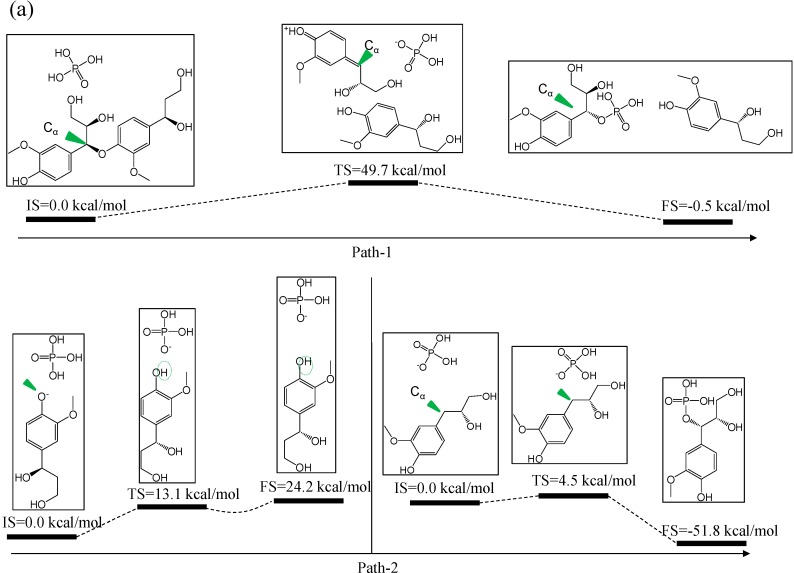

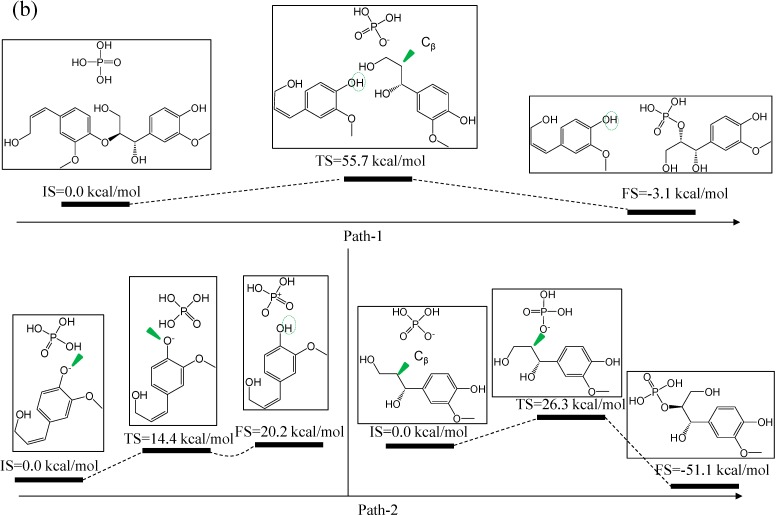
The calculated potential energy profiles for the direct one-step phosphorylation of lignin at (**a**) α*-O-*4 linkage and at (**b**) β*-O-*4 linkage, and the referenced two-step phosphorylation of the decomposed lignin.

Further, we focused on the phosphorylation mechanism of lignin. Figure 3 compares the calculated potential energy profiles for the direct one-step phosphorylation and two-step phosphorylation of β*-O-*4 and α*-O-*4. The one-step reaction channel starts from the optimized adsorption geometries of H_3_PO_4_ on β*-O-*4 and α*-O-*4, respectively.

**Figure 4:**
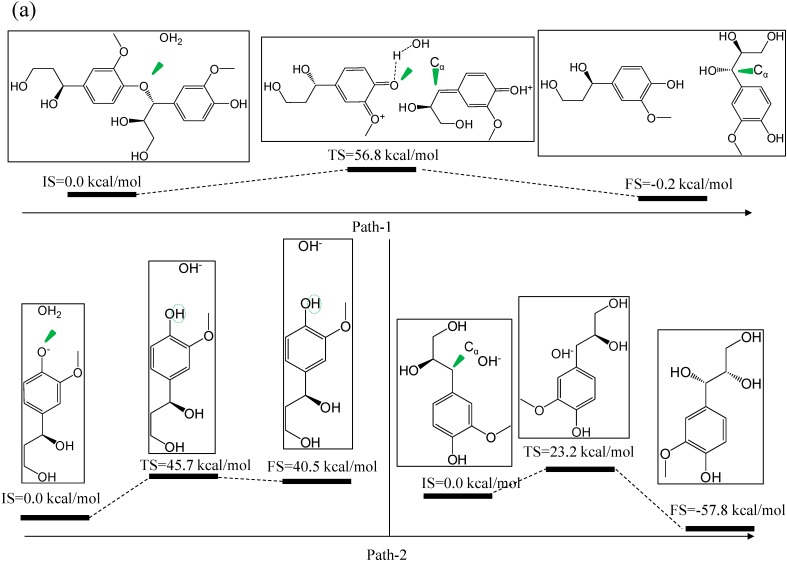

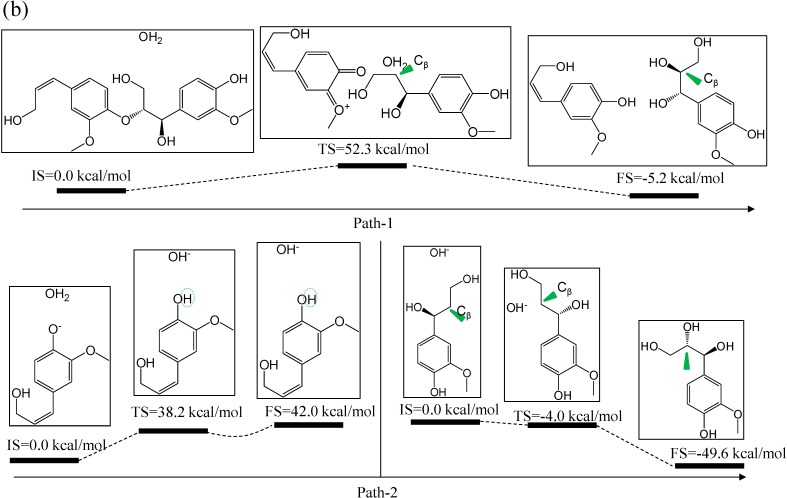
The calculated potential energy profiles for the direct one-step hydrolysis of lignin (**a**) α*-O-*4 linkage and at (**b**) β*-O-*4 linkage, and the referenced two-step hydrolysis of the decomposed lignin.

The two-step reaction channel starts from the stable adsorption of H_3_PO_4_ to the O site (shown with the green arrow in the lower part of Figure 3 and Figure 4) of the decomposed β*-O-*4 and α*-O-*4; then H^+^ from H_3_PO_4_ bonded to the O site of part of the decomposed β*-O-*4 and α*-O-*4 and H_2_PO_4_^−^ bonded to C_β_ and C_α_ of the other part of the decomposed β*-O-*4 and α*-O-*4 at the final state, respectively.

*E*_a_ for the direct one-step phosphorylation of α*-O-*4 is 49.8 kcal/mol, which is higher than that of the decomposition of α*-O-*4 (44.1 kcal/mol shown in Figure 2) in phosphoric acid. Also, the *E*_a_ for the two step mechanism of phosphorylation of α*-O-*4 is far lower than 49.7 kcal/mol, therefore, α*-O-*4 prefers to follow the two-step phosphorylation mechanism based on the decomposition of α*-O-*4 with the relatively lower *E*_a_ of 44.1 kcal/mol.

*E*_a_ for the direct one-step phosphorylation of β*-O-*4 is 55.7 kcal/mol, which is a bit higher than that of the decomposition of β*-O-*4 (55.5 kcal/mol shown in Figure 2). *E*_a_ for the two step mechanism of phosphorylation of β*-O-*4 is far lower than 55.5 kcal/mol. Therefore, β*-O-*4 prefers to follow a two-step phosphorylation mechanism.

According to the calculated *E*_a_, phosphorylation of α*-O-*4 is more energetically accessible than phosphorylation of β*-O-*4 during the treatment of lignin in phosphoric acid. After phosphorylation, H_2_PO_4_^−^ bound to C_β_ and C_α_, respectively, while H^+^ bound the corresponding O atom binding to the aromatic ring of the decomposed α*-O-*4 and β*-O-*4. The stable configuration of H_2_PO_3_-lignin species was obtained after lignin phosphorylation in H_2_PO_3_ solution, which corresponds to the product of the reaction between lignin and phosphorous oxychloride in the presence of pyridine [23].

### 2.3. Hydrolysis of Lignin

In reference to the theoretical study on acid-catalyzed hydrolysis of lignin β*-O-*4 linkages in ionic liquid solvents [24], we focused on the hydrolysis mechanism of lignin. Figure 4 shows the same energy profiles as Figure 3, but for the hydrolysis of lignin. *E*_a_ for the direct one-step hydrolysis of α*-O-*4 is 56.8 kcal/mol, which is higher than that of the decomposition of α*-O-*4 (42.6 kcal/mol shown in Figure 2) in aqueous solution. *E*_a_ for the two-step mechanism of hydrolysis of α*-O-*4 is lower than 56.8 kcal/mol, therefore, α*-O-*4 prefers to follow the two-step hydrolysis mechanism based on the decomposition of α*-O-*4 in aqueous solution.

*E*_a_ for the direct one-step hydrolysis of β*-O-*4 is 52.3 kcal/mol, which is a bit lower than that of the decomposition of β*-O-*4 (55.2 kcal/mol shown in Figure 2). However, though the *E*_a_ for the two-step mechanism of hydrolysis of β*-O-*4 is far lower than 52.3 kcal/mol, the premise is that the two step reactions are based on the decomposition of β*-O-*4 with the *E*_a_ of 55.2 kcal/mol in aqueous solution.

Therefore, β*-O-*4 prefers to follow a one-step hydrolysis mechanism in aqueous solution. After hydrolysis of the α*-O-*4 and β*-O-*4, HO^−^ bonds to C_β_ and C_α_, respectively, while H^+^ bonds to the corresponding O atom linking to the aromatic ring of lignin.

Comparing Figure 3 and Figure 4, we observed that phosphorylation and hydrolysis of α*-O-*4 follow a two-step reaction mechanism, while phosphorylation and hydrolysis of β*-O-*4 follow a one-step reaction mechanism. Due to the relatively small *E*_a_, α*-O-*4 and β*-O-*4 could phosphorylate and hydrolyze in phosphoric acid and aqueous solution, respectively. However, phosphoric acid treatment is the first step of the phosphoric acid-acetone pretreatment process, hence the two-step phosphorylation reaction of α*-O-*4 and the one-step phosphorylation reaction of β*-O-*4 would occur at this acid treatment step. Further, since phosphorylation of α*-O-*4 is more energetically accessible than phosphorylation of β*-O-*4 in phosphoric acid, the phosphorylation of α*-O-*4 could be controllably realized under certain operational condition.

### 2.4. Properties of the Obtained Lignin

**Figure 5:**
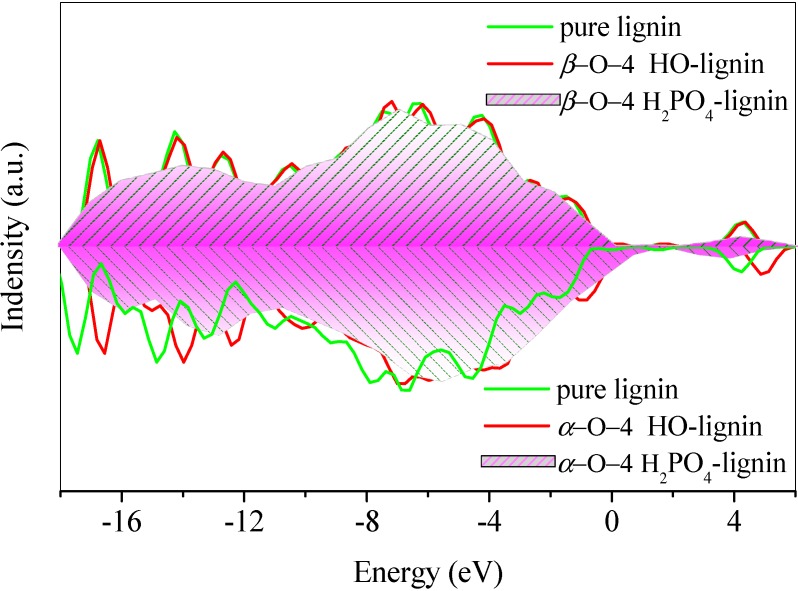
DOS for the pure lignin, HO-functionalized lignin, and the H_2_PO_4_-functionalized lignin.

Hydrolysis and phosphorylation of lignin occur during the phosphoric acid-acetone pretreatment process, which can modify the properties of lignin. For example, -OH groups provide an opportunity to create hydrogen bonds between copolymer and lignin [25]; incorporation of H_2_PO_4_^‒^ group into lignin enhances the adsorption ability of lignin to metal ions [26]. Herein, we revealed how the -OH group and H_2_PO_4_^‒^ group modify the electronic properties of lignin at an atomic level. Figure 5 compares the density of state (DOS) for the pure model lignin containing the most common linkages (shown in Figure 2), the HO-functionalized lignin, and the H_2_PO_4_-functionalized lignin. In comparison with the DOS for the pure lignin and the HO-functionalized lignin, H_2_PO_4_-C_β_ and H_2_PO_4_-C_α_ change the DOS obviously. Electrons of H_2_PO_4_-C_β_ lignin and H_2_PO_4_-C_α_ lignin are highly delocalized near and below the Fermi level. Both hydrolysis and phosphorylation to α*-O-*4 linkage decrease the energy gap between the highest occupied molecular orbit (HOMO) and the lowest unoccupied molecular orbit (LUMO) of lignin.

**Figure 6:**
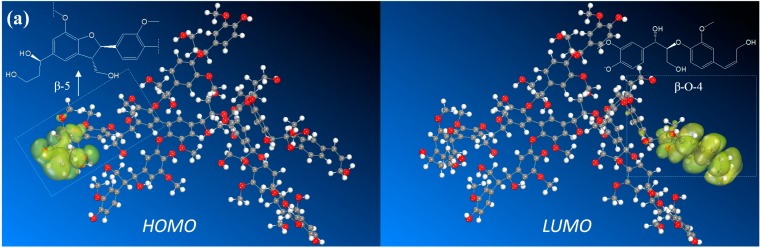

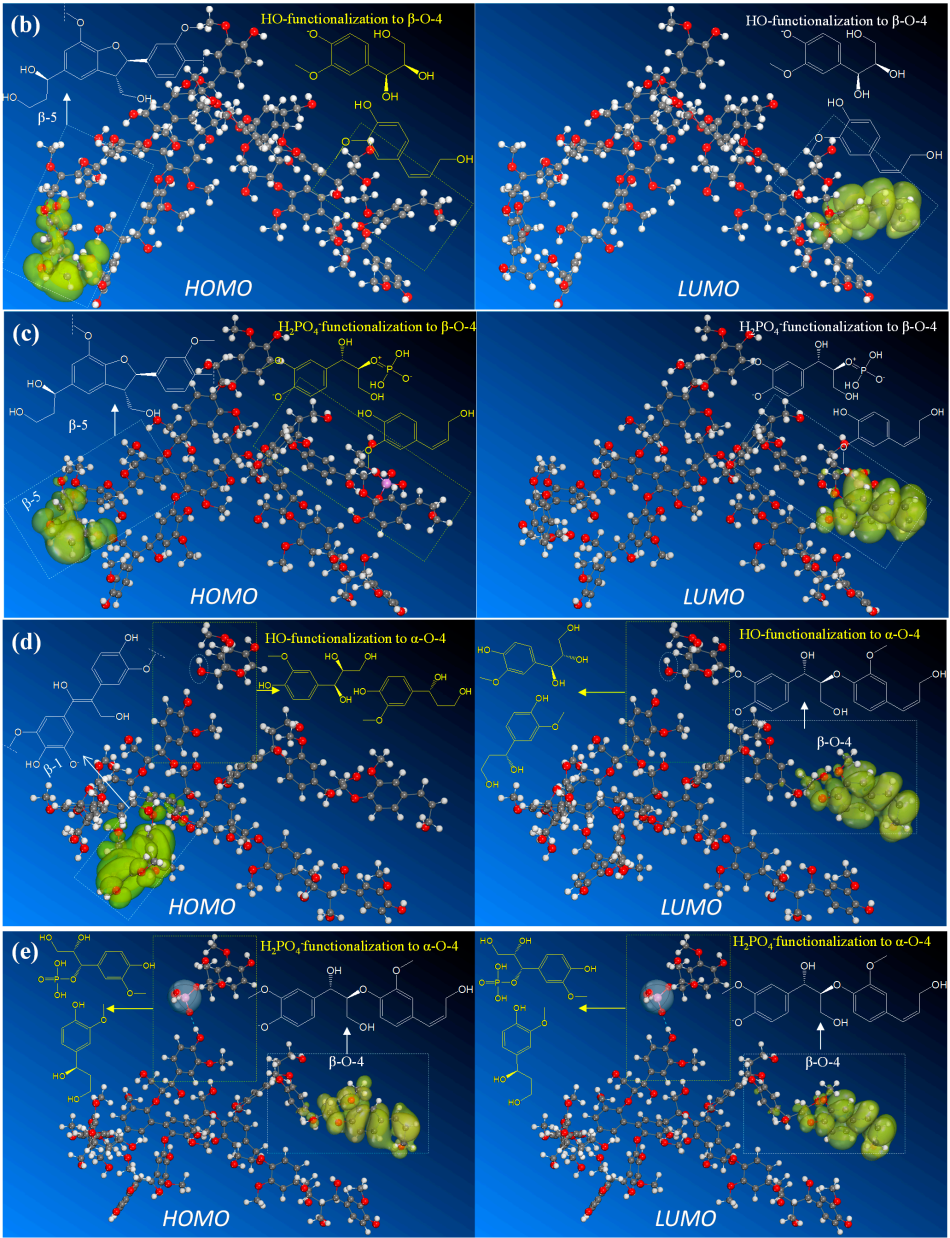
The optimized structure of (**a**) the pure lignin; (**b**) the HO-functionalized lignin at β*-O-*4 linkage; (**c**) the H_2_PO_4_-functionalized lignin at β*-O-*4 linkage; (**d**) the HO-functionalized lignin at α*-O-*4 linkage; and (**e**) the H_2_PO_4_-functionalized lignin at α*-O-*4 linkage, with the isosurface (0.01) of the HOMO and the LUMO.

Further, we compared the HOMO and the LUMO of the lignin before and after hydrolysis and phosphorylation. Figure 6 illustrates the optimized structure of the pure lignin, and the products of phosphorylation and hydrolysis at α*-O-*4 linkage and β*-O-*4 linkage, with the isosurface (0.01) of the related HOMO and LUMO.

In Figure 6a, the left side of β-5 contributes to the HOMO of the pure lignin system, while the right side of β*-O-*4 contributes to the LUMO of the pure lignin system. In Figure 6b,c, the isosurfaces of HOMO and LUMO of the products of phosphorylation and hydrolysis at β*-O-*4 linkage are almost the same as those for the pure lignin in Figure 6a, which suggests that phosphorylation and hydrolysis at β*-O-*4 linkage never change the HOMO and LUMO of lignin.

However, differing from the pure lignin and the H_2_PO_4_-functionalized and HO-functionalized lignin at β*-O-*4 linkage, HO-functionalization at α*-O-*4 linkage changes the HOMO of the lignin system, where β-1 contributes to the HOMO (as shown in Figure 6d), and H_2_PO_4_-functionalization at the α*-O-*4 linkage results in that the right side of β*-O-*4 contributing to boththe HOMO and LUMO of the lignin system (as shown in Figure 6e). Clearly, the phosphorylation of α*-O-*4 in phosphoric acid during the phosphoric acid-acetone pretreatment process of lignin, controls electron and hole transfer on the right side of β*-O-*4 in the functionalized lignin system. Therefore, the right side of β*-O-*4 can act as a special structure with certain functions for novel applications such as lignin-based imprinted polymers, sensors, and molecular devices.

## 3. Computational Details

The equilibrium structure of the model lignin in different solutions was obtained by performing molecular dynamics simulations with the LAMMPS program package [27], using the ReaxFF force field parametrizaion as described in [28]. Based on the equilibrium structure of the model lignin, density functional theory (DFT) calculations were used to investigate the detailed properties of lignin and the related reaction mechanisms in various solutions involved in the phosphoric acid–acetone pretreatment process in reference to the works [29,30,31,32,33,34,35]. In our work, DFT calculations were performed using the DMol3 package. The exchange-correlation energy of electrons was calculated with the spin-polarized generalized gradient approximation (GGA) [36] as implemented in the DMol^3^ package. The Perdew-Burke-Ernzerhof (PBE) exchange-correction functional [37] and the double numerical plus polarization (DNP) [38,39,40] basis set were used throughout the calculations, which is equivalent in accuracy to the commonly used 6-31G** of Gaussian orbital basis set. But the numerical basis set is much more accurate than a Gaussian basis set with the same size. The solvent effect was considered by using a solvation model with the appropriated dielectric constant, ε, where ε was set to be 80.1, 20, and 12.7 for the aqueous solution, acetone, and phosphoric acid (85% *w*/*w*), in reference to the work of Wang [41] and Munson [42]. Specific interactions between lignin and the solvent molecules were not taken into account in this study, since the solvent is represented by a polarizable continuum with a particular dielectric constant. During the calculations, the atoms were relaxed and Brillouin zone integration was performed at the gamma point [43]. Calculations used an energy convergence tolerance of 1 × 10^−6^ Ha and gradient convergence of 1 × 10^−6^ Ha·Å^−1^. A formulation for the linear (LST) and quadratic synchronous transit (QST) methods was used to search the transition states and investigate lignin fragment decomposition [44].

## 4. Conclusions

We theoretically studied the lignin hydrolysis and phosphorylation mechanism during the phosphoric acid–acetone pretreatment process of lignin using density functional theory calculations. The lignin fragment with the seven most common linkages (β*-O-*4, β-β, 4*-O-*5, β-1, 5-5, α*-O-*4, and β-5) was modeled to detect the chemical stability of these linkages in H_3_PO_4_, CH_3_COCH_3_, and H_2_O solutions present during the phosphoric acid–acetone pretreatment process. 5-5, β-1, β-5, β-β, and 4*-O-*5 are chemically stable to maintain the relative structural integrity of lignin, while partial structural damage will happen to lignin due to the relative vulnerability of β*-O-*4 and α*-O-*4. Then two-step phosphorylation reaction happened at α*-O-*4 linkage as the major modification to lignin in H_3_PO_4_ solution, where H_2_PO_4_^−^ group bound to C_α_, resulting in a stable configuration of H_2_PO_3_*-O-*lignin species. H_2_PO_4_-functionalization at α*-O-*4 linkage could controlled electron and hole transfer on the right ring of β*-O-*4 in the H_2_PO_3_*-O-*lignin species. Results provide a fundamental understanding for process-controlled modification of lignin and potential novel applications in lignin-based imprinted polymers, sensors, and molecular devices.
